# Modeling Disease Vector Occurrence when Detection Is Imperfect: Infestation of Amazonian Palm Trees by Triatomine Bugs at Three Spatial Scales

**DOI:** 10.1371/journal.pntd.0000620

**Published:** 2010-03-02

**Authors:** Fernando Abad-Franch, Gonçalo Ferraz, Ciro Campos, Francisco S. Palomeque, Mario J. Grijalva, H. Marcelo Aguilar, Michael A. Miles

**Affiliations:** 1 Instituto Leônidas e Maria Deane – Fiocruz Amazônia, Manaus, Amazonas, Brazil; 2 Pathogen Molecular Biology Unit, Department of Infectious and Tropical Diseases, London School of Hygiene & Tropical Medicine, London, United Kingdom; 3 Biological Dynamics of Forest Fragments Project, Smithsonian Tropical Research Institute/Instituto Nacional de Pesquisas da Amazônia, Manaus, Amazonas, Brazil; 4 Rollins School of Public Health, Emory University, Atlanta, Georgia, United States of America; 5 Centro de Investigación en Enfermedades Infecciosas, Pontificia Universidad Católica del Ecuador, Quito, Ecuador; 6 Tropical Disease Institute, Biomedical Sciences Department, Ohio University College of Osteopathic Medicine, Athens, Ohio, United States of America; 7 Instituto Juan César García – Fundación Internacional de Ciencias Sociales y Salud, Quito, Ecuador; 8 Ministerio de Salud Pública del Ecuador, Quito, Ecuador; Universidad de Buenos Aires, Argentina

## Abstract

**Background:**

Failure to detect a disease agent or vector where it actually occurs constitutes a serious drawback in epidemiology. In the pervasive situation where no sampling technique is perfect, the explicit analytical treatment of detection failure becomes a key step in the estimation of epidemiological parameters. We illustrate this approach with a study of *Attalea* palm tree infestation by *Rhodnius* spp. (Triatominae), the most important vectors of Chagas disease (CD) in northern South America.

**Methodology/Principal Findings:**

The probability of detecting triatomines in infested palms is estimated by repeatedly sampling each palm. This knowledge is used to derive an unbiased estimate of the biologically relevant probability of palm infestation. We combine maximum-likelihood analysis and information-theoretic model selection to test the relationships between environmental covariates and infestation of 298 Amazonian palm trees over three spatial scales: region within Amazonia, landscape, and individual palm. Palm infestation estimates are high (40–60%) across regions, and well above the observed infestation rate (24%). Detection probability is higher (∼0.55 on average) in the richest-soil region than elsewhere (∼0.08). Infestation estimates are similar in forest and rural areas, but lower in urban landscapes. Finally, individual palm covariates (accumulated organic matter and stem height) explain most of infestation rate variation.

**Conclusions/Significance:**

Individual palm attributes appear as key drivers of infestation, suggesting that CD surveillance must incorporate local-scale knowledge and that peridomestic palm tree management might help lower transmission risk. Vector populations are probably denser in rich-soil sub-regions, where CD prevalence tends to be higher; this suggests a target for research on broad-scale risk mapping. Landscape-scale effects indicate that palm triatomine populations can endure deforestation in rural areas, but become rarer in heavily disturbed urban settings. Our methodological approach has wide application in infectious disease research; by improving eco-epidemiological parameter estimation, it can also significantly strengthen vector surveillance-control strategies.

## Author Summary

Blood-sucking bugs of the genus *Rhodnius* are major vectors of Chagas disease. Control and surveillance of Chagas disease transmission critically depend on ascertaining whether households and nearby ecotopes (such as palm trees) are infested by these vectors. However, no bug detection technique works perfectly. Because more sensitive methods are more costly, vector searches face a trade-off between technical prowess and sample size. We compromise by using relatively inexpensive sampling techniques that can be applied multiple times to a large number of palms. With these replicated results, we estimate the probability of failing to detect bugs in a palm that is actually infested. We incorporate this information into our analyses to derive an unbiased estimate of palm infestation, and find it to be about 50% – twice the observed proportion of infested palms. We are then able to model the effects of regional, landscape, and local environmental variables on palm infestation. Individual palm attributes contribute overwhelmingly more than landscape or regional covariates to explaining infestation, suggesting that palm tree management can help mitigate risk locally. Our results illustrate how explicitly accounting for vector, pathogen, or host detection failures can substantially improve epidemiological parameter estimation when perfect detection techniques are unavailable.

## Introduction

Chagas disease is caused by *Trypanosoma cruzi* (Kinetoplastida: Trypanosomatidae), a parasitic protozoan transmitted through the feces of infected blood-sucking hemipterans (Reduviidae: Triatominae) [1],[2]. Human infection is endemic throughout Latin America, where it causes loses of more than 650,000 disability-adjusted life years annually [3]. From 1990, burden figures have declined by about 80% [3],[4], reflecting the success of Chagas disease control programs over vast geographical areas [5]. However, the burden of Chagas disease in the Latin American-Caribbean region is still consistently larger than the *combined* burden of malaria, leprosy, the leishmaniases, lymphatic filariasis, onchocerciasis, schistosomiasis, viral hepatitides B and C, dengue, and the major intestinal nematode infections [6],[7]. Because most transmission is mediated by household-infesting insect vectors, and because no effective treatment or vaccine are available for large-scale use, the elimination of domestic triatomines was defined as one major goal of control programs, together with systematic serological screening of blood donors [8],[9].

The widespread occurrence of native triatomine species that reinvade insecticide-treated households is a major difficulty for the consolidation of Chagas disease control [9]–[12]. Except for a few key vector species (e.g., [13]), the ecological dynamics of reinfestation are still poorly understood, and it is expected that research on sylvatic triatomine populations will help confront the challenge of residual, low-intensity disease transmission mediated by sylvatic vectors. The situation in the Amazon, where enzootic *T. cruzi* transmission cycles involve a great diversity of vectors and reservoir hosts (e.g., [14],[15]), suitably illustrates these concerns. Adventitious adult triatomines maintain continuous, low-intensity transmission in rural (and some urban) settings; as a result, human infection is hypoendemic in the region, with about 100,000 to 300,000 people chronically carrying *T. cruzi* [16],[17]. Sylvatic triatomines are also involved in localized disease outbreaks related to oral *T. cruzi* transmission via contaminated foodstuffs [14],[16], and account for the relatively high infection prevalence (4–5%) reported among extractivist forest workers such as *piaçava* palm fiber collectors [15],[16]. The vast majority of these transmission events are mediated by triatomines of the genus *Rhodnius*, which are primarily associated with palm trees [18]–[20]. The widespread occurrence of palm tree-living *Rhodnius* populations in Amazonia, together with epidemiological evidence suggesting their active role in disease transmission, underscores the importance of obtaining reliable estimates of palm tree infestation rates by these vectors. Such estimates are currently unavailable, and this substantially hinders our understanding of Chagas disease transmission dynamics in the Amazon.

Palms of the genus *Attalea* (Arecoideae) play a major role as breeding and foraging habitats of sylvatic *Rhodnius* populations in Amazonia and other Neotropical regions (e.g., [18]–[23]). The strong *Attalea*-*Rhodnius* association led to the proposal that the presence of *Attalea* palms can be used as an ‘ecological indicator’ of areas where enzootic *T. cruzi* transmission cycles probably occur [23]. Later studies showed that the probabilities of palm infestation by triatomines can differ among sites, landscapes, and palms with varying structural traits [20],[21]. We moved beyond these preliminary proposals, based on limited datasets and crude analytical approaches, and asked under what sets of circumstances is the potential of palms to harbor bug colonies realized; in other words: are all *Attalea* equally likely to be occupied by *Rhodnius* bugs? If not, what are the likely causes of variation? In a region as vast as Amazonia, knowledge of the environmental determinants of palm infestation by triatomines may represent a key tool to optimize resource allocation for epidemiological surveillance. Should resources be aimed at intervention in one particular region, in one particular type of landscape, or on certain particular types of palms – regardless of the region and landscape where they are found? Answers to these questions may prove crucial to enhance disease prevention programs [20],[21].

The estimation of palm infestation by triatomines is limited by the inescapable reality of field sampling: the target organisms may be present at a site yet go undetected during the survey. There are two standard solutions to this pervasive problem. One is to develop improved sampling techniques that bring detection close to perfection. The other is to incorporate detection failure explicitly in the analyses; estimates of infestation can thus be derived that statistically compensate for false absences. Near-perfect sampling techniques are expensive and labor-intensive – clearly a problematic option for a vast study area. In this paper, we apply models developed by wildlife biologists to estimate site-occupancy probabilities when detection of the target organism is imperfect [24],[25]. We define palm infestation as site (i.e., palm) occupancy, the probability that a palm is occupied by at least one *Rhodnius* spp. Our approach leads to strong inferences on *Attalea* palm occupancy rates by *Rhodnius* spp. and allows for the comparison of models relating palm occupancy to environmental covariates at three different scales: region, landscape, and individual palm. We aimed at (i) describing palm infestation patterns and the way they vary at different spatial scales; (ii) identifying the most likely causes of such variation; and (iii) incorporating this information into predictive models of palm occupancy that can be useful in the context of disease risk mitigation. More generally, we illustrate a methodological approach that yields reliable estimates of eco-epidemiological parameters out of imperfect data.

## Methods

### Sampling strategy

**Figure 1:**
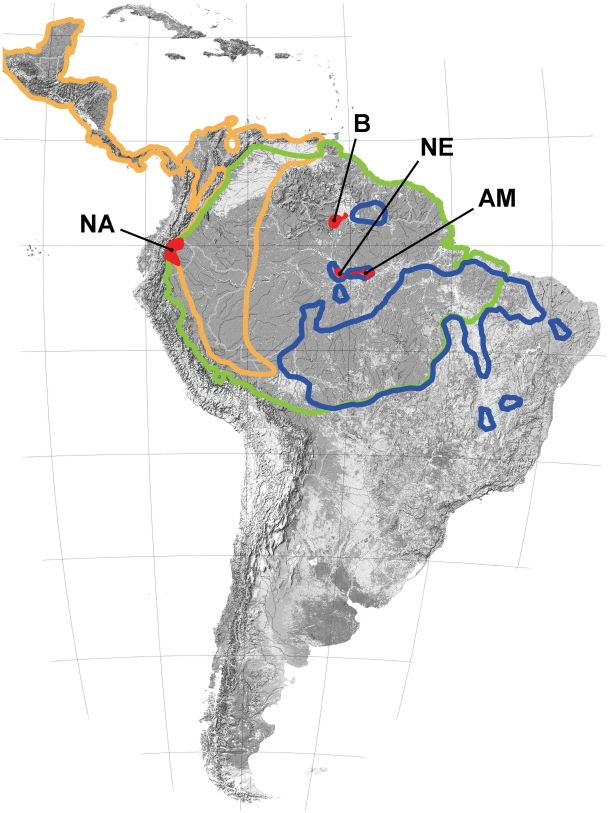
Fieldwork areas and the approximate range (see ref. [29]) of palm tree species investigated for infestation by *Rhodnius* spp.: orange, *Attalea butyracea*; green, *Attalea maripa*; and blue, *Attalea speciosa*. **NA**, Napo region, Ecuador; **NE**, Negro river region, Brazil; **AM**, Amazon river region, Brazil; and **B**, Branco river region, Brazil.

Our sample of 298 *Attalea* palms spanned four regions (totalling 19 localities) in two countries (Fig. 1). The westernmost region was **Napo**, a white-water river system close to the Ecuadorian Andes. (All model covariates are named in **bold** typeface on their first appearance in the Methods section.) Moving to the east, we sampled three regions in the Brazilian Amazon: the lower right bank of the black-water **Negro** river, the left bank of the white-water **Amazon** river east of Manaus, and the forested part of the northern **Branco** river basin, an intermediate clear/white-water system. These survey sites spanned areas between ∼120×60 km (Napo) and ∼30×20 km (Negro), and were located, respectively, within each of the following moist forest ecoregions [26]: Napo, Japurá/Solimões-Negro, Uatumã-Trombetas, and Guyanan Highlands/Piedmont. From field observations and available literature [27],[28], we ranked our survey regions in decreasing order of soil fertility as Napo, Amazon, Negro, and Branco. Thus, or sampling is representative of four ecologically distinct sub-regions influenced by the three main Amazonian hydrological systems – white-, black-, and clear-water.

Within each region, we surveyed *Attalea* palms in three landscape classes: **forest**, **rural**, and **urban**. At each site, a sample of non-adjacent palms was selected haphazardly for the survey. Urban palms where sampled in plots within the street framework of cities, towns, or villages. Rural palms were surrounded by farming land, orchards, or pasture on previously forested sites. Forest palms were located in forested sites, most often medium to large fragments of mature secondary forest. These three landscape classes were easily distinguished in the field, and palms sampled in each of them were at least 50–100 m from the nearest patch of landscape in another class. Our sample included palms of three species (*A. maripa*, *A. speciosa*, and *A. butyracea*); their known distribution is shown in Fig. 1. All three species are large, solitary palms with large inflorescences/infructescences and in which old leaf bases remain adhered to the stem after leaf abscission. Palm identification followed Henderson et al. [29].

### Palm traits

Individual palm trees vary considerably with regard to the amounts of epiphytic vegetation and dead organic material (dead fronds, husks, flowers, fruits, fibers, and dead epiphytes) that accumulate on their crowns and stems. We used a pre-established score system [21] to measure the approximate amount of live epiphytic plants and decomposing organic material present on each palm. These epiphyte and organic matter values were first recorded in the field and, for about 85% of palms, cross-checked by another team member by examination of individual palm photographs; we then derived a mean ‘**organic score**’ value for each palm – ranging from 0 to 4 points, with higher values denoting ‘dirtier’ palms. We measured palm stem **height** as the linear distance between the ground and the lowest base of a green leaf. Finally, we preliminarily assessed the effects of slash-and-burn farming practices, which are commonplace across the Brazilian Amazon, on palm infestation. We defined two coarse categories to distinguish palms standing on plots that had a **fire** less than about two years before our survey from palms on plots that were not burnt over a similar period. Fire information was obtained from landowners and complemented by recording fire scars on palms and nearby trees and the presence and size of fire-adapted pioneer trees in each survey plot.

### Detecting infestation

**Figure 2:**
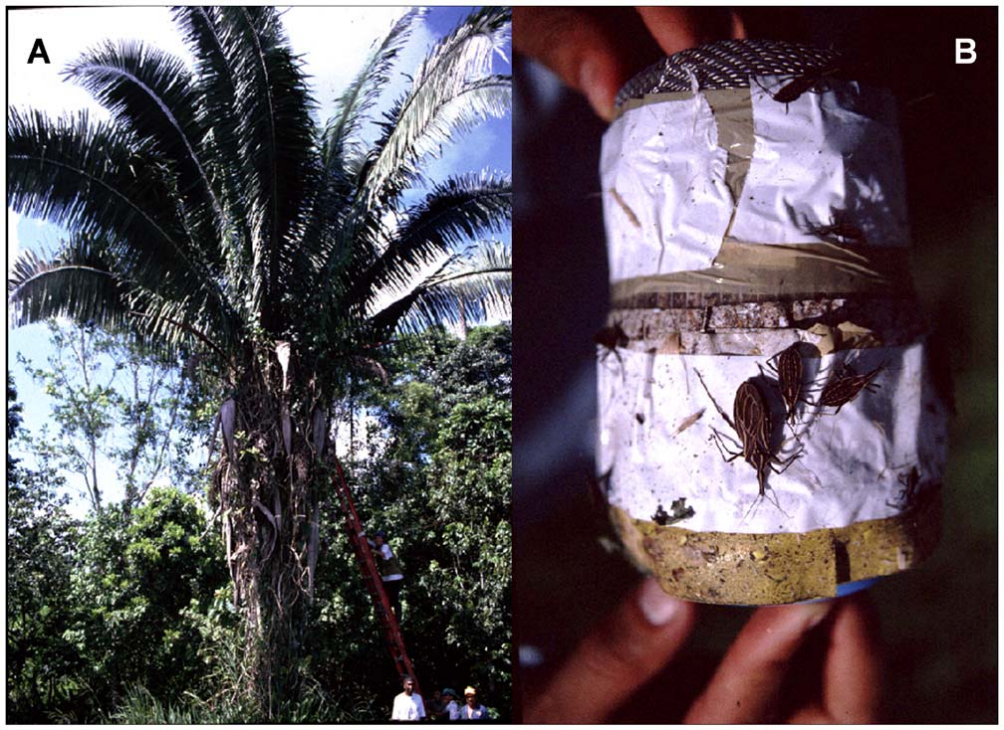
Sampling *Rhodnius* spp. in *Attalea* palm trees. **A**: a ladder is used to climb an *Attalea butyracea* palm to remove traps and manually search for bugs. **B**: a mouse-baited adhesive trap with several *Rhodnius* specimens adhered to the tape.

We sampled each individual palm with a combination of mouse-baited adhesive **traps** [30],[31] and **manual** bug searches [32] (Fig. 2). Traps were set in the afternoon and checked the following morning, after approximately 15 hours of operation. We placed traps among organic debris or epiphytes in the palm crown, around the upper end of the stem, or directly in the angle between palm fronds. Most palms (234, or 78.5%) were sampled with four traps, with a minimum of one trap in eight palms and a maximum of nine in one palm. The total trapping effort was 1,098 trap-nights. Manual searches were performed on the organic matter of the palm crown after trap removal. We searched either directly in the palm crown or by collecting organic material in a 50-liter plastic bag and later checking bag contents on a white canvas. Both sampling techniques were used in 255 palms (85.6%), only manual searches in nine, and only traps in 34. Each individual trap or manual search was treated as a sampling event yielding a binary result of either “1” for bug detection or “0” for no bugs detected. Thus, a typical palm tree was sampled five times – four traps and one manual search. Each detection history is represented by a row of “1”s and “0”s. For instance, “1100-----0” represents a palm with two positive traps, two negative traps, and a negative manual search (the last “0”); the five dashes indicate that only four traps, up to a maximum of nine, were operated in this particular palm. The raw dataset is provided as Supporting Information (Dataset S1).

### Data analysis

We combine two different but interconnected procedures: parameter estimation and model selection. All our models have a biological process component that expresses the probability that a palm is occupied by bugs (ψ), and a sampling process component that expresses the probability that we detect bugs in a palm where they actually occur (*p*). This hierarchical approach makes it possible to estimate the probability that animals are present in places where they are not seen, accommodating an explicit treatment of imperfect detection [24],[25],[33],[34]. We fit models using the software PRESENCE [35], which provides maximum-likelihood estimates of parameters and their standard errors (SE) in user-defined models that can contain covariates of occupancy and/or detection. Before performing the analyses, we built a set of 23 models (below) each expressing an *a priori* hypothesis of palm occupancy and bug detection. Model selection followed the Akaike Information Criterion (AIC), which combines information and maximum-likelihood theories to find models with the best compromise between model fit and complexity [36]. We use model selection as a tool for hypothesis testing: each model represents one hypothesis, and hypotheses represented by models with lower AIC values are better supported by the data.

### Model structure

We treat palms as independent sites with regard to occupancy by bugs of the genus *Rhodnius*; to ensure independence, several sites were surveyed within each locality, and neighboring palms were rarely sampled. Live-bait traps and manual searches are treated as replicate sampling events with an average probability of detecting bugs, conditioned on palm occupancy. Field observations and exploratory analyses motivated us to compare the performance of manual searches and traps in detecting bugs; furthermore, we observed relatively high numbers of triatomines per palm in the Napo region, suggesting that bug presence might be easier to detect in Napo palms than elsewhere. Accordingly, we modeled detection always as an additive logistic function of two binary covariates: sampling technique and region, with the latter specifying only whether sampling took place in Napo or elsewhere. Since we aimed at understanding which spatial scale contributes most to explaining observed variation in palm occupancy, we built models that include different palm, landscape, and regional covariates of occupancy. Our *a priori* set of 23 models includes six regional models, four landscape models, six local (palm) models, six models with different combinations of covariates from different scales, and one null model without covariates of occupancy. Some of the combined models include interactions between covariates at different scales. In particular, considering the more fertile soils of the Napo region, we model an interaction between Napo and the rural landscape, as well as between Napo and the forest landscape. These models represent hypotheses stating that the relationship between landscape and occupancy differs between Napo and the remaining regions. For ease of presentation, we will report modeling results grouped by spatial scale, concluding with a comparison of the best models across scales.

## Results

### Null model

We first estimated detection probability with a simple model that has no covariates of palm occupancy. We designate this model with the notation ‘ψ(.), *p*(manual+Napo)’, where the ‘.’ denotes no covariates on the occupancy part of the model and ‘manual’ and ‘Napo’ designate the technique and regional covariates of detection, respectively. Under this null model of no predictable variation in palm occupancy rates, the probability of detecting bugs where they actually occur ranges from 0.05 (SE = 0.01) with traps in the Brazilian Amazon to 0.82 (SE = 0.05) with manual searches in Napo, Ecuador. Both covariates increase detection probabilities; the Napo effect estimate is 3.01 (SE = 0.3). Had we not taken detection failure into account, we would report a proportion of 0.24 palms occupied by bugs – the number of palms where we detected bugs divided by the total number of palms sampled, which when expressed as a percentage is the classical ‘infestation index’ [9] (Table 1). When we consider that the probability of detection may be less than one, our null model estimate of occupancy is 0.59 (CI_95%_ 0.42–0.75).

**Table 1:** *Rhodnius* spp. in *Attalea* spp. palm trees in Amazonia: Entomological indices and characteristics of 298 palms surveyed in four geographical-ecological regions.

Variable	Region*				Total
	Napo	Negro	Amazon	Branco	
Coordinates**	0°25′S 77°00′W	2°50′S 60°55′W	3°05′S 59°00′W	2°25′S 61°05′W	
Palms sampled [infested]	46 [26]	87 [14]	85 [19]	80 [13]	298 [72]
Infestation index (%)	56.5	16.1	22.4	16.3	24.2
Infestation index, traps (%)	51	9.2	14.1	12.2	18
Infestation index, manual searches (%)	100	7.9	16.5	7.5	14
Bugs captured	235	24	59	20	338
Bugs/palms sampled (M±SD)	5.11±10.4	0.28±0.8	0.69±2.6	0.25±0.7	1.13±4.6
Bugs/infested palms (M±SD) [Md, Max]	9±12.5 [4.5, 56]	1.7±1.1 [1, 4]	3.1±4.7 [2, 22]	1.5±1.2 [1, 5]	4.7±8.5 [2, 56]
Trap-nights	137	345	341	275	1098
Traps/palms sampled (M)	3	4	4	3.4	3.7
Palm stem height (M) [CI_95%_], in m	7.2 [6.7–7.7]	6 [5.6–6.4]	6.4 [6–6.7]	6.4 [6–6.8]	6.4 [6.2–6.6]
Palms in recently burned land	0	10	11	9	30
Organic score (M) [CI_95%_]	2 [1.75–2.16]	1.8 [1.69–1.99]	1.7 [1.62–1.88]	1.6 [1.47–1.73]	1.8 [1.69–1.84]
Organic score (Md)	2	1.75	1.75	1.5	1.75
Palms sampled (forest/rural/urban)	21/17/8	28/48/11	5/42/38	22/42/16	76/149/73

*xsAs defined in the text.

**Approximate geographic coordinates of the central area of each study region.

M = mean; SD = standard deviation; Md = median; Max = maximum; CI_95%_ = 95% confidence interval.

### Regional models

We found little evidence of regional variation in occupancy, as shown by the small differences in AIC values between the null model and models with regional covariates (Table 2). When we constrain models to only one regional covariate, the region that contributes most to explaining the data is Napo. All the models that estimate occupancy in the Napo region separately from other regions set that value at 0.68 (CI_95%_ 0.50–0.83), almost twice the average occupancy estimated for Brazilian regions (0.37; CI_95%_ 0.22–0.54). The second model in Table 2 includes regional covariates for the two hypothetical extremes of occupancy, Napo and Branco. Despite our prior expectation, based on published soil richness information, this model does not explain the data any better than the single-covariate Napo model. Thus, even if the Napo region appears to have higher palm occupancy rates, the data do not provide strong evidence of variation in occupancy across regions, and in particular among regions within Brazil.

**Table 2:** Regional-scale models of *Attalea* palm occupancy by *Rhodnius* spp. in four sampling areas in Amazonia.

Model	*k*	ΔAIC	*w*_i_	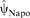	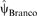	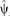
ψ(Napo), *p*(manual+Napo)	5	0	0.399	0.68±0.09	-	0.37±0.08
ψ(Napo+Branco), *p*(manual+Napo)	6	1.87	0.157	0.68±0.09	0.34±0.11	0.38±0.09
ψ(.), *p*(manual+Napo)	4	2.29	0.127	-	-	0.59±0.09
ψ(Negro), *p*(manual+Napo)	5	2.71	0.103	-	-	0.60±0.08
ψ(Region), *p*(manual+Napo)	7	2.83	0.097	0.68±0.09	0.34±0.11	-
ψ(Branco), *p*(manual+Napo)	5	3.61	0.066	-	0.48±0.15	0.60±0.09
ψ(Amazon), *p*(manual+Napo)	5	4.11	0.051	-	-	0.59±0.09

Models include different combinations of covariates of *Attalea* palm occupancy by *Rhodnius* spp. at the regional scale. Model structure and covariates are defined in the Methods section. ‘Region’ denotes a model where all four regions differ from each other in occupancy; 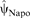 and 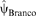 show occupancy estimates for the Napo and Branco regions, respectively. We show these two regions only because they represent extremes of soil fertility. 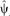 gives an average estimate of occupancy probability that applies to all regions not named as covariates of occupancy; thus, the exact meaning of 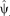 changes between models. In the models of occupancy in individual regions, 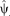 represents the average occupancy probability across all regions. ΔAIC is the variation in Akaike Information Criterion values relative to the best model (in first row); *w*_i_ is the Akaike weight, a normalized likelihood of the model; and *k* is the number of model parameters.

### Landscape models

Estimated palm occupancy is highest in rural and lowest in urban settings, without striking differences between estimates for different landscapes (Table 3). The models with interaction terms (Napo*forest and Napo*rural) do not explain the data particularly better than models without those terms. Among models with only one landscape covariate, the best model estimates a negative effect of urban landscapes on occupancy and lumps rural and forest areas into one landscape class. Estimated palm infestation rates are 0.33 (CI_95%_ 0.15–0.57) for urban and 0.63 (CI_95%_ 0.45–0.78) for forest/rural landscapes. Despite these broad patterns, there is no strong evidence of landscape-level effects: AIC values vary within less than 10 units for all models, and there is overlap of 95% CIs for estimates of occupancy in different landscapes.

**Table 3:** Landscape-scale models of *Attalea* palm occupancy by *Rhodnius* spp. in four sampling areas in Amazonia.

Model	*k*	ΔAIC	*w*_i_	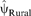	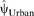	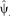
ψ(Ld+Napo*forest), *p*(manual+Napo)	8	0	0.374	0.42±0.09	0.17±0.07	-
ψ(Region+Ld), *p*(manual+Napo)	9	1.66	0.163	-	-	-
ψ(urban), *p*(manual+Napo)	5	1.91	0.144	-	0.33±0.11	0.63±0.09
ψ(Ld+Napo*rural), *p*(manual+Napo)	8	2.21	0.124	0.43±0.10	0.20±0.08	-
ψ(Ld), *p*(manual+Napo)	6	3.13	0.078	0.69±0.12	0.34±0.12	-
ψ(rural), *p*(manual+Napo)	5	3.43	0.067	0.72±0.12	-	0.51±0.09
ψ(.), *p*(manual+Napo)	4	4.71	0.035	-	-	0.59±0.09
ψ(forest), *p*(manual+Napo)	5	6.68	0.013	-	-	0.61±0.11

Models include different combinations of covariates of *Attalea* palm occupancy by *Rhodnius* spp. at the landscape scale. Model structure and covariates are defined in the Methods section. ‘Ld’ designates a model where all three landscape classes have different occupancies, while ‘Region+Ld’ denotes the full additive occupancy model with all regions and all landscapes. The operator ‘*’ indicates an interaction between regional and landscape covariates. The notation 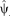 shows estimates of ψ that apply to all landscape classes not mentioned in the occupancy model name; its exact meaning changes between models. ΔAIC is the variation in Akaike Information Criterion values relative to the best model (in first row); *w*_i_ is the Akaike weight, a normalized likelihood of the model; and *k* is the number of model parameters.

### Local models

All the models that include the ‘organic score’ palm attribute perform substantially better than the null model (Table 4). We modeled the effects of organic score, height, and recent fire separately and in two additive combinations (all effects and the combination of height and organic score) after preliminary analyses suggested that recent fire was the least important of the three covariates. AIC variation across models indicates that height and organic score are indeed most useful to explain the data. A model with all covariates does not rank any better than the model with height and organic score alone. When the three covariates are modeled separately, organic score ranks better than height, which, in turn, ranks better than fire. The strength of these relationships between infestation and individual palm traits is at odds with expectations under random bug migration among palms within a given site, indicating that the assumption of palm independence with regard to occupancy holds.

**Table 4:** Local-scale models of *Attalea* palm occupancy by *Rhodnius* spp. in four sampling areas in Amazonia.

Model	*k*	ΔAIC	*w*_i_
ψ(score+height), *p*(manual+Napo)	6	0	0.473
ψ(Lc), *p*(manual+Napo)	7	0.80	0.317
ψ(Ld+Lc), *p*(manual+Napo)	9	2.58	0.130
ψ(R+Lc), *p*(manual+Napo)	10	4.84	0.042
ψ(R+Ld+Lc), *p*(manual+Napo)	12	5.44	0.031
ψ(score), *p*(manual+Napo)	5	10.06	0.003
ψ(score+fire), *p*(manual+Napo)	6	10.50	0.003
ψ(height), *p*(manual+Napo)	5	14.01	0.001
ψ(fire), *p*(manual+Napo)	5	25.58	0.000
ψ(.), *p*(manual+Napo)	4	26.27	0.000

Models include different combinations of covariates of *Attalea* palm occupancy by *Rhodnius* spp. at the local scale. Model structure and covariates are defined in the Methods section. ‘Lc’, ‘Ld’, and ‘R’ stand for the full additive models of palm attributes (score, height, and fire), landscape, and region, respectively. The occupancy model ‘R+Ld+Lc’ combines additive effects from all spatial scales. ΔAIC is the variation in Akaike Information Criterion values relative to the best model (in first row); *w*_i_ is the Akaike weight, a normalized likelihood of the model; and *k* is the number of model parameters.

### Cross-scale comparisons

**Figure 3:**
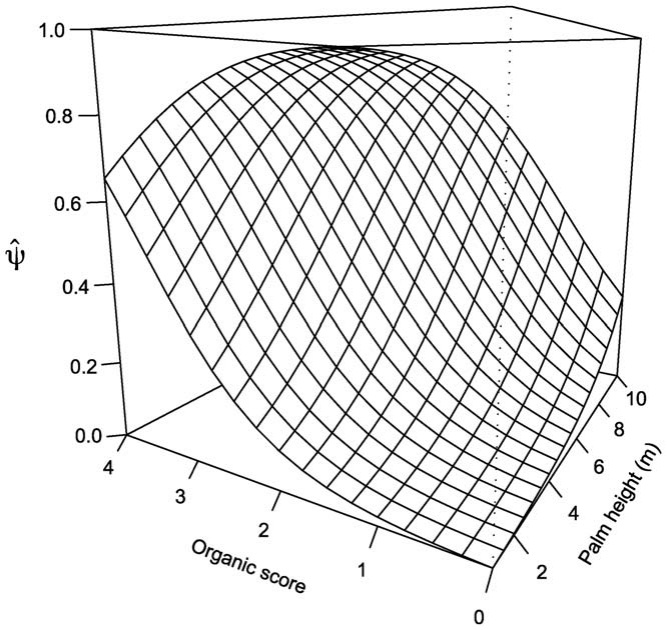
Estimates of *Attalea* palm tree occupancy by *Rhodnius* spp. as a function of palm tree height and organic score under the best performing model.

Tables 2 and 3 show how regional and landscape models fall within less than 10 AIC units of the null model, suggesting that they do not improve our ability to explain the data when compared with a model lacking occupancy covariates. Conversely, Tables 4 and 5 show strong support for local-scale models that use palm attributes as covariates of occupancy. Models that include regional and/or landscape covariates jointly with palm attributes also perform substantially better than the null model. However, these multi-scale models do not explain the data any better than a simple local model of occupancy as a function of organic score and palm height – the first model of Tables 4 and 5, where both effects are positive and significantly larger than zero (1.41, SE = 0.41; and 0.43, SE = 0.13, respectively). Figure 3 shows occupancy estimates according to this best-performing model. Short and ‘clean’ *Attalea* palms have the lowest probability of infestation, whereas tall palms (∼10 m) with plenty of accumulated organic debris are predicted to be almost certainly infested. According to these point estimates of occupancy by *Rhodnius* spp., a ‘clean’ palm would have, at most, a 0.3 probability of infestation; this probability would rise to over 0.5 in a palm with an organic score close to 4. Parameter estimates for the best-ranking models are provided as Supporting Information (Table S1).

**Table 5:** The complete set of 23 *a priori* models of *Attalea* palm occupancy by *Rhodnius* spp. in four sampling areas in Amazonia: cross-scale comparisons.

Model	Scale	AIC	ΔAIC	*w*_i_	*k*
ψ(score+height), *p*(manual+Napo)	Lc	594.86	0.00	0.4732	6
ψ(Lc), *p*(manual+Napo)	Lc	595.66	0.80	0.3172	7
ψ(Ld+Lc), *p*(manual+Napo)	Lc+Ld	597.44	2.58	0.1303	9
ψ(R+Lc), *p*(manual+Napo)	Lc+R	599.70	4.84	0.0421	10
ψ(R+Ld+Lc), *p*(manual+Napo)	Lc+Ld+R	600.30	5.44	0.0312	12
ψ(score), *p*(manual+Napo)	Lc	604.92	10.06	0.0031	5
ψ(score+fire), *p*(manual+Napo)	Lc	605.36	10.50	0.0025	6
ψ(height), *p*(manual+Napo)	Lc	608.87	14.01	0.0004	5
ψ(Ld+Napo*forest), *p*(manual+Napo)	Ld*R	616.42	21.56	0.0000	8
ψ(R+Ld), *p*(manual+Napo)	Ld+R	618.08	23.22	0.0000	9
ψ(urban), *p*(manual+Napo)	Ld	618.33	23.47	0.0000	5
ψ(Ld+Napo*rural), *p*(manual+Napo)	Ld*R	618.63	23.77	0.0000	8
ψ(Napo), *p*(manual+Napo)	R	618.84	23.98	0.0000	5
ψ(Ld), *p*(manual+Napo)	Ld	619.55	24.69	0.0000	6
ψ(rural), *p*(manual+Napo)	Ld	619.85	24.99	0.0000	5
ψ(fire), *p*(manual+Napo)	Lc	620.44	25.58	0.0000	5
ψ(Napo+Branco), *p*(manual+Napo)	R	620.71	25.85	0.0000	6
ψ(.), *p*(manual+Napo)	(null)	621.13	26.27	0.0000	4
ψ(Negro), *p*(manual+Napo)	R	621.55	26.69	0.0000	5
ψ(R), *p*(manual+Napo)	R	621.67	26.81	0.0000	7
ψ(Branco), *p*(manual+Napo)	R	622.45	27.59	0.0000	5
ψ(Amazon), *p*(manual+Napo)	R	622.95	28.09	0.0000	5
ψ(forest), *p*(manual+Napo)	Ld	623.10	28.24	0.0000	5

Models include different combinations of covariates of *Attalea* palm occupancy by *Rhodnius* spp. at the local (Lc), landscape (Ld), and regional (R) scales. Model structure and covariates are defined in the Methods section. ‘R’ appears as a covariate of occupancy in models where occupancy differs among all four regions, ‘Ld’ in models where occupancy differs among all three landscape classes, and ‘Lc’ in models with occupancy varying as a function of palm attributes (organic score, stem height, and fire). The operators ‘+’ and ‘*’ indicate additive models and models with interactions, respectively. AIC is the Akaike Information Criterion; ΔAIC is the variation in AIC relative to the best model (in first row); *w*_i_ is the Akaike weight, a normalized likelihood of the model; and *k* is the number of model parameters. All models above the dotted line include both palm organic score and stem height as covariates of palm occupancy; all models above the dashed line include organic score as a covariate of occupancy. The null model (with no covariates of occupancy) is identified as “(null)”.

## Discussion

A coherent view of the epidemiology of Chagas disease in Amazonia is currently emerging; discrete foci of relatively intense transmission, related to large-scale harvesting or consumption of forest products, seem to punctuate a widespread background pattern of low-intensity, vector-borne transmission [15]–[17]. Faced with the logistical impossibility of full geographical coverage, surveillance systems rely on a combination of two strategies: (i) detection of acute, febrile cases of the disease through existing health services (malaria posts and the regular health care network), and (ii) identification of higher-risk areas or situations that can be targeted through localized control and prevention efforts [37]. The first strategy is limited by the low sensitivity of clinical diagnosis [2],[38]; the detection of *T. cruzi* in malaria blood smears depends on the levels of parasitemia and requires skilled technicians. The second approach demands a clear understanding of the environmental circumstances that signal a higher risk of disease transmission. We focus on this second option, using the quantification of vector occurrence as a proxy for epidemiological risk and modeling palm occupancy by vectors as a function of environmental covariates over three spatial scales. To the best of our knowledge, this is the first attempt to develop quantitative models relating environmental factors to the occurrence of triatomine vectors in Amazonia.

Had we measured palm infestation as the percentage of palms where bugs were detected [9], we would report an infestation index of 24.2% (72 out of 298 palms; Table 1). Instead, we explicitly considered the possibility that bug detection fails in some palms that are actually infested, and derived an unbiased estimate of palm occupancy that is twice as high as the classical infestation index. This hierarchical strategy of modeling occupancy and detection as separate but inter-related processes stems from methods developed for estimating animal population parameters under imperfect detection [25],[33],[34], and is particularly useful when target organisms are of small size, dull-colored, and secretive (see Box 1). Many human disease vectors match this description, and most triatomine species surely do. Vector population studies that disregard the imperfections of the sampling process are likely to yield biased conclusions that may result in flawed recommendations for disease control and surveillance [see 39,40].

**Box 1. Modeling Occupancy under Imperfect Detection: Practical Guidelines**

1. **Defining an occupancy problem.** Ensure that the study system is usefully portrayed as a set of spatially discrete sampling units (e.g., households, persons) that may or may not be occupied by the organism of interest (e.g., infested, infected) at a given time.
2. **Is imperfect detection involved?** Estimating occupancy with imperfect detection makes sense only if there is a non-negligible chance that the target organism is not seen in a sampling unit where it actually occurs (i.e., get ‘false-negative’ results). Detection failure may not become apparent until the same unit is repeatedly sampled; in practice, most organisms are detected imperfectly.
3. **Temporal scope of replication.** If the goal is estimating occupancy at one point in time, sampling units must not change their occupancy status during the sampling period. To ensure the fulfillment of this “closure” assumption, repeated sampling must take place within a sufficiently short time-frame that will depend on the mobility of the target organism relative to sampling units. When temporal variation is of interest, replication in pre-defined short periods across years or seasons must follow the same rules as the single-period sampling. For detailed guidelines on sampling design, see ref. [62].
4. **Model specification.** Models must embody alternative hypothetical, plausible explanations of the biological data and sampling process at hand. Each model is specified as a combination of covariates that can influence occupancy and/or detection probabilities. The analyses will identify which hypothetical explanation is best supported by the data.
5. **Model selection.** The Akaike Information Criterion (AIC) is frequently used for model selection; it favors the best compromise between model fit to the data and simplicity of the hypothetical explanation as measured by the number of model parameters [36],[63]. In our case, model selection was instrumental in understanding the importance of local environmental factors to palm occupancy by triatomine bugs.
6. **Parameter estimation.** Te final step is to estimate the parameters for each model. We did this in a maximum-likelihood framework as described in refs. [24],[25]. Our approach is easily implemented using PRESENCE [35], where you can estimate occupancy and detection parameters as well as the magnitude of covariate effects. For complex problems requiring more analytical flexibility, a Bayesian framework may be preferable [53]. Royle and Dorazio [33] provide a comprehensive introduction to Bayesian hierarchical analyses; the free R and WinBUGS software packages implement these methods.

It must be noted that environmental constraints not included in our analyses could also modify palm occupancy. For instance, bug populations are under the influence of seasonality, predation pressure, and host availability. The efficacy of live-bait traps may vary with the nutritional status of the bugs, their aggressiveness or the performance of adhesive tapes under different weather conditions. Thus, while our models provide a simple and informative explanation of the data at hand, a more detailed assessment of triatomine population ecology and *T. cruzi* transmission dynamics in Amazonia will require the measurement and analysis of additional covariates.

Our data contain indirect information on vector abundance that is reflected in the estimates of detection probability [41]. The high estimates of detection probabilities in the Napo region (∼0.55 *vs.* ∼0.08 elsewhere) match our field observation of relatively larger numbers of bugs per occupied palm (9.04 *vs.* 2.24 in Brazil); this suggests a possible relation between soil fertility and bug density, perhaps mediated by higher primary productivity in rich-soil ecosystems. Whether this relation holds and has any public health relevance in other Amazonian fertile-soil regions is still an open question. It must be noted, however, that the prevalence of human *T. cruzi* infection in the Ecuadorian Amazon, including our Napo survey area, is substantially higher (2.4%) than the overall estimate (∼1%) for the whole Amazon basin [16],[17]. Such difference warns against using palm occupancy as the sole metric of transmission risk, and calls for further research to test the soil fertility-vector abundance hypothesis. Studies of vector abundance should also investigate how the number of bugs in a palm relates to the probability that adult specimens fly into a nearby house [42]. There is evidence that in denser triatomine colonies each individual has less access to bloodmeals, and that adult bugs are more likely to start dispersive flights when starved [43],[44], but the data are still inconclusive for sylvatic *Rhodnius* populations.

The effects of anthropogenic habitat disturbance on triatomine bug populations have been discussed extensively (e.g., [9],[20],[42],[45]); however, the evidence to support the claim that habitat disturbance triggers house invasion or colonization by triatomines is still weak. Our results show similar palm occupancy rates in forest and rural areas, but lower occupancy in urban settings. This suggests that palm tree *Rhodnius* populations can endure moderate habitat degradation, including slash-and-burn farming, in deforested rural areas, but tend to become rarer in heavily disturbed urban landscapes. Such endurance may sustain the risk of vector-human contact in rural sites, particularly when selective deforestation respects large palm trees near houses – a common practice across the Neotropics. We caution that our observations about urban landscapes may not apply directly to large urban forest fragments or to the contact zones between forests and expanding urban settlements; triatomines are known to occur in these environments, and may regularly enter houses near forest edges (e.g., [19],[46]).

Our data provide substantial support to previous observations suggesting that individual palm tree attributes have a strong influence on infestation probabilities [20],[21]. The mechanisms underlying this phenomenon have not been thoroughly investigated; we hypothesize that larger and ‘dirtier’ palms constitute better micro-environments for the bugs in terms of both structural traits and host availability. High organic score values translate into higher architectural complexity, resulting in more hiding and oviposition sites, and probably help maintain stable and buffered microclimate conditions [cf. 20]. The number of potential vertebrate hosts available as bloodmeal sources for the bugs can also be expected to be higher in larger palms with higher organic score values [47], where more hiding/nesting sites, and often also fruits and seeds, are available. Our hypothesis predicts that a *Rhodnius* population infesting a large, dirty palm tree has less chances of going extinct than a population infesting a small, clean palm. This hypothesis may be tested with a patch occupancy dynamics study [48].

### Conclusions

This paper highlights the importance of accounting for imperfect detection in the study of vector ecology; in addition, our assessment of the explanatory power of regional, landscape, and local environmental covariates aimed at identifying those that hold more promise for improving vector surveillance and control strategies [49],[50].

Our results are relatively discouraging with regard to broad-scale risk mapping; the use of soil richness datasets seems attractive, but prior validation studies are necessary. On the other hand, local-scale covariates are overwhelmingly more useful than regional or landscape features in explaining variations in palm occupancy. This suggests that the assessment of potential disease risk situations will require detailed knowledge of local, site-specific conditions. The participation of decentralized vector control teams linked to local malaria control services [16],[37] may therefore be key to the advancement of Chagas disease prevention in Amazonia. Our results also suggest that peridomestic palm tree management could lower palm infestation rates and, therefore, might help reduce transmission risk [21]. Model-predicted effects of removing organic debris from palms range from halving to reducing palm infestation probability by more than 70% (Fig. 3). This result indicates correlation, not necessarily causation, but provides a clear-cut working hypothesis that can be put to test in the context of environmental management research.

Imperfect detection of the target organism is a real and pervasive problem both in wildlife management and in epidemiology. Wildlife biologists often use sampling strategies (e.g., [51]) and analytical tools [52],[53] that yield unbiased parameter estimates under imperfect detection. Latent class analysis and capture-recapture approaches are used to formally account for detection failure in epidemiological studies; they allow estimation of prevalence or incidence rates when a diagnostic gold standard is unavailable or undercount of disease events is likely (e.g., [54]–[58]). Even if the contribution of these and similar approaches is growing, we still find that many epidemiological and most vector ecology studies simply overlook the problem of imperfect detection.

Here we show how replicate sampling of vector ecotopes with a practical, yet imperfect field methodology can be used to (i) derive unbiased statistical estimates of eco-epidemiological parameters and (ii) test hypotheses about the effects of environmental covariates on such parameters. As long as model assumptions (e.g., population closure or independent detection histories) hold reasonably and study design is adequate, this strategy can help enhance research on vectors, pathogens, and hosts (see Box 1). For instance, replicate malaria blood smears could be used to measure between-slide variation in *Plasmodium* spp. detection. The same reasoning applies to vector surveillance schemes with replicate sampling, e.g., of *Aedes aegypti* [59], or when pathogen diagnosis involves serial testing, e.g., for intestinal parasites [60]. The generality of our methodological proposal is particularly compelling in the case of vector-borne zoonotic diseases, which are those more likely to become emerging public health threats [61], but the formal treatment of imperfect detection can significantly strengthen other areas of eco-epidemiological research.

## Acknowledgments

A. Paucar, C. Carpio, R. Perry, and technicians of Fiocruz and the Ecuadorian and Brazilian vector control services participated in fieldwork. We thank T.V. Barrett (INPA, Brazil), C.J. Schofield (LSHTM and ECLAT, UK), F. Noireau (IRD, Bolivia), and S.L.B. Luz (ILMD-Fiocruz, Brazil) for helpful discussion and suggestions. The Brazilian Instituto Nacional de Colonização e Reforma Agrária provided logistic support for several field trips. This paper is contribution number 9 of the Research Program on Infectious Disease Ecology in the Amazon (RP-IDEA) of the Instituto Leônidas e Maria Deane.

## Supporting Information

Alternative Language Abstract S1 Spanish translation of the abstract by FA-F.
(0.03 MB DOC)

Alternative Language Abstract S2 Portuguese translation of the abstract by FA-F.
(0.03 MB DOC)

Dataset S1 Occupancy of 298 Amazonian palm trees by triatomine bugs: Raw dataset.
(0.12 MB XLS)

Table S1 Effects of covariates on *Attalea* palm occupancy by *Rhodnius* spp. and on bug detection probability: parameter estimates for the seven best-ranking models as assessed with the Akaike Information Criterion. Effect size, sign, and standard error are given for each covariate in the corresponding model.
(0.04 MB DOC)
