# Supplementary material for: Modeling Disease Vector Occurrence when Detection Is Imperfect: Infestation of Amazonian Palm Trees by Triatomine Bugs at Three Spatial Scales
Source: PLoS Negl Trop Dis. 2010 Mar 2;4(3):e620. doi: 10.1371/journal.pntd.0000620 (PMC2830460; doi:10.1371/journal.pntd.0000620)
Supplement: Alternative Language Abstract S1 — Spanish translation of the abstract by FA-F. (0.03 MB DOC) [file pntd.0000620.s001.doc]

**Spanish translation of the Abstract by Fernando Abad-Franch**

**Resumen**

***Introducción.*** La no detección de un patógeno o vector en un lugar donde de hecho está presente constituye un serio problema en epidemiología. Cuando no existen técnicas de muestreo perfectas, lo que es extremadamente común, el tratamiento analítico explícito de los fallos de detección se convierte en un paso fundamental de la estimación de parámetros epidemiológicos. Este enfoque es ilustrado con un estudio de infestación de palmeras *Attalea* por *Rhodnius* spp. (Triatominae), los principales vectores de la enfermedad de Chagas en el norte de Sudamérica.

***Métodos/Hallazgos Principales.*** Estimamos la probabilidad de detectar triatominos en palmeras infestadas por medio del muestreo repetido de cada palmera. Este conocimiento es utilizado para derivar una estimación no sesgada de la probabilidad biológicamente relevante de infestación de palmeras. Combinando estimación de parámetros por máxima verosimilitud y selección de modelos por criterios derivados de la teoría de la información, evaluamos las relaciones entre covariables ambientales e infestación de 298 palmeras amazónicas en tres escalas espaciales: región dentro de la Amazonia, paisaje y palmera individual. Las estimaciones de infestación son altas (40-60%) en todas las regiones, muy por encima de la tasa observada de infestación (24%). Las probabilidades de detección son más altas (~0.55 en promedio) en la región con suelos más ricos que en el resto (~0.08). Las tasas de infestación son similares en áreas de bosque y rurales, pero más bajas en zonas urbanas. Finalmente, las covariables individuales de cada palmera (cantidad de residuos orgánicos y altura del tronco) explican la gran mayoría de la variación de las tasas de infestación.

***Conclusiones/Importancia.*** Las características individuales de las palmeras aparecen como el principal determinante de la infestación, lo que sugiere que la vigilancia de la enfermedad de Chagas deberá incorporar conocimiento de las condiciones locales y que el manejo de palmeras peridomésticas puede ayudar a reducir el riesgo de transmisión. Las poblaciones de vectores son probablemente más densas en regiones con suelos más ricos, que es donde la prevalencia de la enfermedad tiende a ser más elevada; esto sugiere un objetivo para la investigación sobre mapeamiento del riesgo en escala regional. Los efectos en la escala del paisaje indican que las poblaciones de triatominos que ocupan palmeras pueden tolerar la deforestación en zonas rurales, pero se vuelven más raras en áreas urbanas fuertemente alteradas. La propuesta metodológica que presentamos tiene aplicaciones mucho más amplias en la investigación sobre enfermedades infecciosas; al mejorar la estimación de parámetros eco-epidemiológicos, la propuesta puede también fortalecer las estrategias de control y vigilancia de vectores.
