# Supplementary material for: Modeling Disease Vector Occurrence when Detection Is Imperfect: Infestation of Amazonian Palm Trees by Triatomine Bugs at Three Spatial Scales
Source: PLoS Negl Trop Dis. 2010 Mar 2;4(3):e620. doi: 10.1371/journal.pntd.0000620 (PMC2830460; doi:10.1371/journal.pntd.0000620)
Supplement: Alternative Language Abstract S2 — Portuguese translation of the abstract by FA-F. (0.03 MB DOC) [file pntd.0000620.s002.doc]

**Portuguese translation of the Abstract by Fernando Abad-Franch**

**Resumo**

***Introdução.*** A não detecção de um agente patogênico ou vetor num local onde de fato ele está presente constitui um sério problema em epidemiologia. Quando não existem técnicas de amostragem perfeitas, o que é extremamente comum, o tratamento analítico explícito das falhas de detecção vira um passo fundamental da estimativa de parâmetros epidemiológicos. Esta abordagem é ilustrada com um estudo de infestação de palmeiras *Attalea* por *Rhodnius* spp. (Triatominae), os principais vetores da doença de Chagas no norte da América do Sul.

***Métodos/Achados Principais.*** Estimamos a probabilidade de detectar triatomíneos em palmeiras infestadas por meio da amostragem repetida de cada palmeira. Este conhecimento é utilizado para derivar uma estimativa não enviesada da probabilidade biologicamente relevante de infestação de palmeiras. Combinando estimativa de parâmetros por máxima verossimilhança e seleção de modelos por critérios derivados da teoria da informação, avaliamos as relações entre covariáveis ambientais e infestação de 298 palmeiras amazônicas em três escalas espaciais: região dentro da Amazônia, paisagem e palmeira individual. As estimativas de infestação são altas (40-60%) em todas as regiões, muito acima da taxa observada de infestação (24%). As probabilidades de detecção são mais altas (~0.55 em média) na região com solos mais ricos do que no resto (~0.08). As taxas de infestação são similares em áreas de floresta e rurais, mas menores em zonas urbanas. Finalmente, as covariáveis individuais de cada palmeira (quantidade de resíduos orgânicos e altura do caule) explicam a grande maioria da variação das taxas de infestação.

***Conclusões/Importância.*** As características individuais das palmeiras aparecem como o principal determinante da infestação, o que sugere que a vigilância da doença de Chagas deverá incorporar conhecimento das condições locais e que o manejo de palmeiras peridomésticas pode ajudar a reduzir o risco de transmissão. As populações de vetores são provavelmente mais densas em regiões com solos mais ricos, onde a prevalência da doença tende a ser mais elevada; isto sugere um objetivo para a pesquisa sobre mapeamento do risco em escala regional. Os efeitos na escala da paisagem indicam que as populações de triatomíneos que ocupam palmeiras podem tolerar o desmatamento nas áreas rurais, mas ficam mais raras em áreas urbanas fortemente alteradas. A proposta metodológica que apresentamos tem aplicações muito mais amplas na pesquisa sobre doenças infecciosas; ao melhorar a estimativa de parâmetros eco-epidemiológicos, a proposta pode também fortalecer as estratégias de controle e vigilância de vetores.
