# Supplementary material for: Modeling Disease Vector Occurrence when Detection Is Imperfect: Infestation of Amazonian Palm Trees by Triatomine Bugs at Three Spatial Scales
Source: PLoS Negl Trop Dis. 2010 Mar 2;4(3):e620. doi: 10.1371/journal.pntd.0000620 (PMC2830460; doi:10.1371/journal.pntd.0000620)
Supplement: Table S1 — Effects of covariates on Attalea palm occupancy by Rhodnius spp. and on bug detection probability: parameter estimates for the seven best-ranking models as assessed with the Akaike Information Criterion. Effect size, sign, and standard error are given for each covariate in the corresponding model. (0.04 MB DOC) [file pntd.0000620.s004.doc]

**Table S1. Effects of covariates on *Attalea* palm occupancy by *Rhodnius* spp. and on bug detection probability: parameter estimates for the seven best-ranking models as assessed with the Akaike Information Criterion. Effect size, sign, and standard error are given for each covariate in the corresponding model.**

| **Model*** | **Effects on occupancy** | | | | | | | | **Effects on detection** | |
| --- | --- | --- | --- | --- | --- | --- | --- | --- | --- | --- |
|  | **Regional scale covariates**** | | | **Landscape scale covariates**** | | **Local scale covariates** | | |  | |
|  | Negro | Amazon | Branco | Rural | Urban | Score | Height | Fire | Manual | Napo |
| ψ(score+height), *p*(manual+Napo) | - | - | - | - | - | 1.41±0.41 | 0.43±0.13 | - | 1.49±0.27 | 2.75±0.30 |
| ψ(score+height+fire), *p*(manual+Napo) | - | - | - | - | - | 1.39±0.40 | 0.42±0.13 | -0.97±0.88 | 1.50±0.27 | 2.73±0.30 |
| ψ(Ld+score+height+fire), *p*(manual+Napo) | - | - | - | 0.27±0.54 | -0.63±0.65 | 1.29±0.40 | 0.42±0.13 | -0.83±0.86 | 1.51±0.28 | 2.71±0.30 |
| ψ(R+score+height+fire), *p*(manual+Napo) | -1.10±0.71 | -0.58±0.64 | -0.62±0.73 | - | - | 1.31±0.39 | 0.34±0.13 | -0.80±0.85 | 1.55±0.29 | 2.49±0.35 |
| ψ(R+Ld+score+height+fire), *p*(manual+Napo) | -1.31±0.69 | -0.52±0.66 | -0.89±0.68 | 0.30±0.50 | -0.76±0.64 | 1.19±0.38 | 0.31±0.12 | -0.68±0.82 | 1.58±0.29 | 2.43±0.35 |
| ψ(score), *p*(manual+Napo) | - | - | - | - | - | 1.59±0.47 | - | - | 1.46±0.27 | 2.86±0.30 |
| ψ(score+fire), *p*(manual+Napo) | - | - | - | - | - | 1.50±0.45 |  | -1.18±0.91 | 1.47±0.27 | 2.82±0.30 |

*See main text and Table 5 for details on model structure; **Regional and landscape effects are relative to the omitted categories at each scale: Napo and forest, respectively
